# Myocardial performance index in female athletes

**DOI:** 10.1186/s12947-017-0112-9

**Published:** 2017-09-11

**Authors:** Zahraa Alsafi, Andreas Malmgren, Petri Gudmundsson, Martin Stagmo, Magnus Dencker

**Affiliations:** 1 Department of Medical Imaging and Physiology, Skåne University Hospital, Lund University, Malmö, Sweden; 2 Department of Biomedical Science, Malmö University, Malmö, Sweden; 3 Department of Cardiology, Skåne University Hospital, Lund University, Lund, Sweden

**Keywords:** Athlete’s heart, Diastolic function, Echocardiography, Left ventricle, Myocardial performance index, Right ventricle, Systolic function

## Abstract

**Background:**

Long-term intensive training leads to morphological and mechanical changes in the heart generally known as “athlete’s heart”. Previous studies have suggested that the diastolic and systolic function of the ventricles is unaltered in athletes compared to sedentary.

The purpose of this study was to investigate myocardial performance index (MPI) by pulsed wave Doppler (PWD) and by tissue Doppler imaging (TDI) in female elite athletes compared to sedentary controls.

**Methods:**

The study consisted of 32 athletes (mean age 20 ± 2 years) and 34 sedentary controls (mean age 23 ± 2 years). MPI by PWD and TDI were measured in the left (LV) and right ventricle (RV) in both groups. Moreover, comparisons of MPI by the two methods and between the LV and RV within the two groups were made.

**Results:**

There were no significant differences in MPI between athletes and controls (*p* > 0.05), whereas the LV had significantly higher MPI compared to RV (*p* < 0.001, in athletes and controls). The agreement and the correlation between the two methods measuring MPI showed low agreement and no correlation (athletes RV *r* = −0.027, LV *r* = 0.12; controls RV *r* = 0.20, LV *r* = 0.30).

**Conclusion:**

The global function of the LV and RV measured by MPI with PWD and TDI is similar in female athletes compared to sedentary controls. Conversely, both MPI by PWD and by TDI shows a significant difference between the LV and RV. However, the agreement and correlation between conventional methods of measuring MPI by PWD compared to MPI by TDI is very poor in both these populations.

## Background

Persistent vigorous training increases the need for oxygenated blood to metabolic tissues in the body leading to morphological and mechanical changes in the heart generally known as “athlete’s heart” [1–9]. The function of the athlete’s heart has not been extensively studied, with regard to different sports and female gender. Moreover, the function of the left ventricle (LV) has been examined more than the right ventricle (RV) [9–12].

Sports can be divided into different categories but can be classified in three main groups; dynamic, static or combined (dynamic and static) sports [4, 9]. Morphologic changes of the athlete’s heart are related to the type of sport practised by the athlete but it is also related to gender [6].

Myocardial performance index (MPI) is an easily measured index for the assessment of global heart function, combining both systolic and diastolic components. It can be derived from both pulsed wave Doppler (PWD) and tissue Doppler imaging (TDI) and is defined as the sum of the isovolumic contraction time (ICT) and the isovolumic relaxation time (IRT) divided by the ejection time (ET) [10, 13–24]. MPI has been shown to be independent of HR, blood pressure, loading conditions and the geometry of the ventricles [11, 17, 20, 21] and can be used to evaluate the function of both the RV and the LV [22].

No studies up till now have, to our knowledge, investigated MPI in both RV and LV in female athletes. For this reason, the aim of this study was to gain further understanding of the function of female athlete’s heart by evaluating differences in conventional MPI and MPI by TDI in elite female team-handball players compared to a sedentary group of females.

## Methods

### Study population

The study consisted of 35 female elite team-handball players (mean age 20 ± 2 years) and 34 sedentary controls (mean age 23 ± 2 years). Of the 35 handball players, two were excluded because of cardiovascular disease, one because of a bicuspid aortic valve and one because of a recent hospitalization of suspected myocarditis. Another handball player was excluded due to insufficient quality of echocardiographic image recording. Hence, the final study population included a total of 32 female elite team-handball players. The females included in the control group did not perform any or only slight physical activity, less than two hours a week and were a similar age to the handball players. Weight and height were measured with participants dressed in light clothing. Body mass index (BMI) was calculated as the weight in kilograms divided by the height in meters squared (kg/m^2^). The formula by Du Bois and Du Bois [25] was used for the calculation of BSA. Systolic (SBP) and diastolic (DBP) blood pressure was measured in a supine position in the right arm after 10 min rest (Omron M8 Comfort, Omron Healthcare, Kyoto, Japan). All participants were given a written questionnaire to define their amount of training during a week. The female elite handball players trained in average 10.8 ± 2.3 h per week, whereas 1.7 h were strength training and another 1.8 h per week were fitness training. The remaining of the training time was used for team-game handball training. Two of the participants in the control group were smokers, while none of the athletes were smokers. All participants gave their written informed consent to take part in the study. The study was approved by the Regional Ethical Review Board of Lund University in Sweden.

### Echocardiography

Participants were examined using an iE33 (Philips Medical systems, Andover, MA, USA) ultrasound equipment, with an S3 transducer, according to current guidelines by American Society of Echocardiography [26] as previously described [27]. The study was performed with the participants resting in a left lateral decubitus position. All measurements were performed three times on separate cardiac cycles and averaged. One experienced echocardiographer performed all examinations and measurements were performed off-line by another single observer using Xcelera (Philips Medical systems, Andover, MA, USA).

### Conventional pulsed wave Doppler

To derive MPI of RV (MPIRV) by conventional PWD, an apical four-chamber view was obtained and the sample volume was placed between the tips of the leaflets of the tricuspid valve. An interval “a” was measured from the end to the onset of tricuspid inflow and it represents the sum of ICT, IRT and ET. The right ventricular ejection time (RVET) was obtained from the short-axis view and the sample volume was placed below the pulmonary valve. The interval “b” that represents the RVET was measured from the onset to the end of the RV outflow.

MPI of LV (MPILV) was measured from the apical four-chamber view by placing the sample volume at the tips of the mitral valve leaflets. From the end to the onset of mitral inflow “a” interval was measured. From the apical five-chamber view with the sample volume placed below the aortic valve, “b” interval was measured between onset and end of LV outflow. MPI was later calculated as (a-b)/b, representing (ICT + IRT)/ET [26]. The mean differences of MPI by PWD and TDI were tested between the two groups. Moreover, MPILV and MPIRV were tested within each group.

### Tissue Doppler imaging

**Fig. 1:**
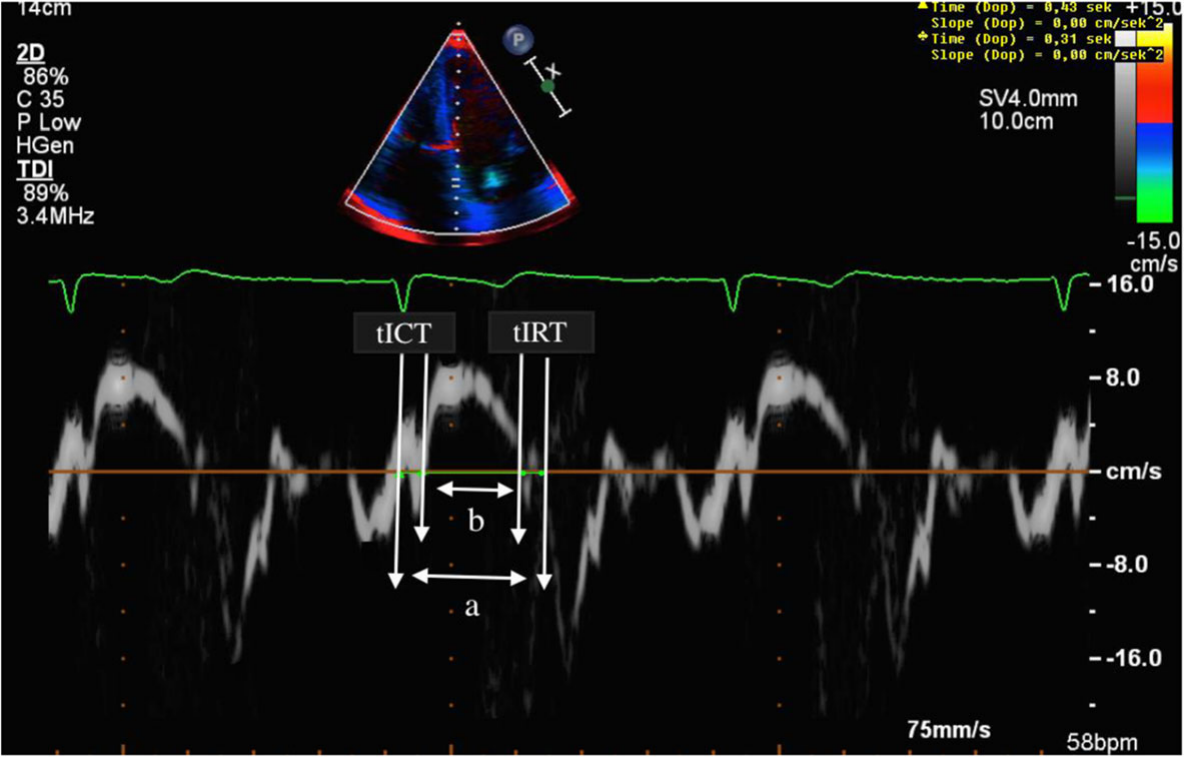
Myocardial performance index measured by tissue Doppler imaging. Time intervals by tissue Doppler imaging derived from septal annulus. tICT, tissue isovolumic contraction time; tIRT, tissue isovolumic relaxation time; a = isovolumic contraction time + isovolumic relaxation time + ejection time; b = ejection time. MPI = (a-b)/b

MPI by TDI (TDMPI) was obtained from the apical four-chamber view by placing the sample volume at the lateral mitral annulus, lateral tricuspid annulus and septal annulus. Measurements of TDI isovolumic contraction time (tICT) were obtained by measuring from the end of a′-wave (atrial-contraction wave) to the onset of s-wave (myocardial systolic wave); TDI isovolumic relaxation time (tIRT) was obtained by measuring between the end of the S-wave and the onset of the e′-wave (early-diastolic wave); TDI ejection time (tET) was measured from onset to the end of s-wave (Fig. 1). TDMPI was then calculated as (tICT + tIRT)/tET [26]. A mean value was additionally calculated between TDMPI at lateral mitral annulus (TDMPIlm) and TDMPI at septal annulus (TDMPIs) that was named (mTDMPIm/s), and also between TDMPI at lateral tricuspid annulus (TDMPIlt) and TDMPIs, (mTDMPIt/s).

A comparison was made between all TDMPI parameters, where TDMPIlm was compared to TDMPIs and to TDMPIlt. Furthermore, TDMPIs was compared to TDMPIlt and finally mTDMPIm/s compared to mTDMPIt/s. The comparisons were made within each group but also the same comparisons were made in both groups combined.

Because the echocardiographic measurements were performed by one observer, intra variability measurements were made in 5 subjects in the athletes group and 5 subjects in the sedentary control group. The intra variability measurements in the athlete group in MPIRV was 7,6%, MPILV 9,2%, TDMPIlm 6,4%, TDMPIs 7,3%, TDMPIlt 4,4%, TDMPIm/s 5% and TDMPIt/s 5,6%. In the sedentary group MPIRV was 8%, MPILV 3,4%, TDMPIlm 3,7%, TDMPIs 2,8%, TDMPIlt 3,2%, TDMPIm/s 1,6% and TDMPIt/s was 2,0%.

### Statistical analysis

All statistical analyses were performed using standard statistical software (SPSS version 22.0, Inc., Chicago, IL, USA). Data are expressed as mean ± standard deviation (SD). The data was tested for normal distribution, by a visual analysis of histograms and by a Q-Q plot, which showed a normal distributed material. The independent Student’s t-test was used to test for mean differences between the two groups. The paired Student’s t-test was used to test for mean differences between different parameters within a group. Bland-Altman plots were used to test the agreement between the conventional MPI by PWD and TDMPI. The correlation between the two methods was tested with Pearson’s correlation coefficient. Values were considered statistically significant at a *p*-value below 0.05.

## Results

The acquired images were of good quality in all subjects, which made all planned measurements possible. All demographics and subject characteristics are presented in Table 1. The study population consisted of totally 66 participants, 32 athletes and 34 sedentary controls. Age, length, weight, BMI, BSA, DBP and HR were significantly different between the two groups. There were no significant differences in SBP and MPI data between athletes and controls.

**Table 1:** Demographics and echocardiographic data of the athletes and the sedentary controls

	Athletes (*n* = 32)	Sedentary (*n* = 34)	*p*-value
Age (years)	20.4 ± 2.0	23.2 ± 1.7	< 0.001
Length (cm)	175 ± 7	171 ± 5	0.02
Weight (kg)	73.1 ± 8.0	64.3 ± 8.5	< 0.001
BMI (kg/m^2^)	23.9 ± 2.1	22.0 ± 2.8	0,002
BSA (m^2)^	1.88 ± 0.13	1.75 ± 0.12	< 0.001
Heart rate (beats/min)	56 ± 8	70 ± 13	< 0.001
SBP (mm Hg)	121 ± 8	121 ± 11	ns
DBP (mm Hg)	67 ± 6	71 ± 7	0.004
MPIRV	0.23 ± 0.13	0.20 ± 0.12	ns
MPILV	0.35 ± 0.07	0.32 ± 0.08	ns
TDMPIlm	0.47 ± 0.06	0.49 ± 0.08	ns
TDMPIs	0.52 ± 0.07	0.54 ± 0.09	ns
TDMPIlt	0.44 ± 0.07	0.45 ± 0.08	ns
mTDMPIm/s	0.50 ± 0.05	0.51 ± 0.07	ns
mTDMPIt/s	0.48 ± 0.06	0.50 ± 0.07	ns

Data values are presented as mean ± SD

Body mass index (BMI), Body surface area (BSA), Diastolic blood pressure (DBP), Systolic blood pressure (SBP), Mean value of tissue Doppler myocardial performance index derived from lateral mitral annulus and septum (mTDMPIm/s), Mean value of tissue Doppler myocardial performance index derived from lateral tricuspid annulus and septum (mTDMPIt/s), Conventional myocardial performance index of left ventricle (MPILV), Conventional myocardial performance index of right ventricle (MPIRV), Myocardial performance index by tissue Doppler of lateral mitral annulus (TDMPIlm), Myocardial performance index by tissue Doppler of lateral tricuspid annulus (TDMPIlt), Myocardial performance index by tissue Doppler of septum (TDMPIs)

MPIRV compared to MPILV showed a significant difference in the athletes group as well as in the sedentary group. The results are displayed in Table 2.

**Table 2:** Myocardial performance index derived from conventional pulsed Doppler in athletes and sedentary

Participant	MPIRV	MPILV	*p*-value
Athlete (n = 32)	0.23 ± 0.13	0.35 ± 0.07	< 0.001
Sedentary (n = 34)	0.20 ± 0.12	0.32 ± 0.08	< 0.001

Data values are presented as mean ± SD

Conventional myocardial performance index of left ventricle (MPILV). Conventional myocardial performance index or right ventricle (MPIRV)

All TDMPI parameters were compared to each other in both groups and all parameters showed a significant difference except for two in the athletes group, TDMPIlm compared to TDMPIlt. Likewise, TDMPIm/s compared to TDMPIt/s in the athletes group did not show any difference. When both athletes group and sedentary group were put together, all parameters showed a significant difference. All results are displayed in Tables 3 and 4.

**Table 3:** Comparison of myocardial performance index by tissue Doppler in athletes and sedentary

Tissue Doppler MPI	Athletes: (n = 32)	Sedentary (n = 34)
	Mean ± SD	Mean ± SD
A) TDMPIlm	0.47 ± 0.06	0.49 ± 0.08
B) TDMPIs	0.52 ± 0.07	0.54 ± 0.09
C) TDMPIlt	0.44 ± 0.07	0.45 ± 0.08
D) mTDMPIm/s	0.50 ± 0.05	0.51 ± 0.07
E) mTDMPIt/s	0.48 ± 0.06	0.50 ± 0.07

Data values are presented as mean ± SD

Mean value of tissue Doppler myocardial performance index derived from lateral mitral annulus and septum (mTDMPIm/s), Mean value of tissue Doppler myocardial performance index derived from lateral tricuspid annulus and septum (mTDMPIt/s), Myocardial performance index by tissue Doppler of lateral mitral annulus (TDMPIlm), Myocardial performance index by tissue Doppler of lateral tricuspid annulus (TDMPIlt), Myocardial performance index by tissue Doppler of septum (TDMPIs)

Athletes: A vs B *p* = 0.001, A vs C *p* = 0.16, B vs C *p* = < 0.001, D vs E *p* = 0.15. Sedentary: A vs B *p* = 0.001, A vs C *p* = 0.045, B vs C *p* = < 0.001, D vs E *p* = 0.03

**Table 4:** Comparison of myocardial performance index by tissue Doppler in the combined group of athletes and sedentary controls (*n* = 66)

Tissue Doppler MPI	Mean ± SD
A) TDMPIlm	0.48 ± 0.07
B) TDMPIs	0.53 ± 0.08
C) TDMPIlt	0.45 ± 0.08
D) mTDMPIm/s	0.50 ± 0.06
E) mTDMPIt/s	0.49 ± 0.07

Data values are presented as mean ± SD

Mean value of tissue Doppler myocardial performance index derived from lateral mitral annulus and septum (mTDMPIm/s), Mean value of tissue Doppler myocardial performance index derived from lateral tricuspid annulus and septum (mTDMPIt/s), Myocardial performance index by tissue Doppler of lateral mitral annulus (TDMPIlm), Myocardial performance index by tissue Doppler of lateral tricuspid annulus (TDMPIlt), Myocardial performance index by tissue Doppler of septum (TDMPIs)

A vs B *p* = < 0.001, A vs C *p* = 0.01, B vs C *p* = < 0.001, D vs E *p* = 0.01

**Fig. 2:**
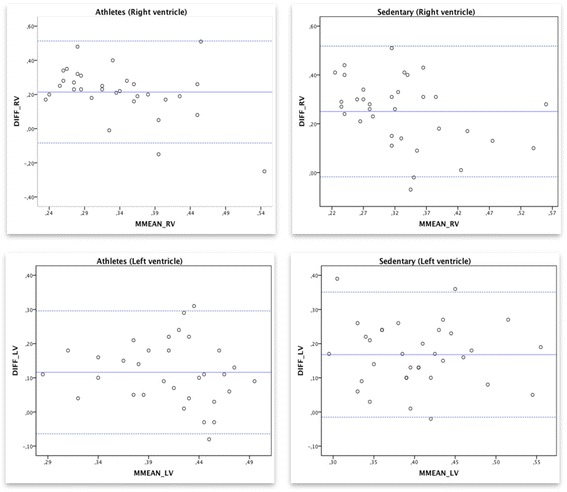
Bland-Altman plot for conventional myocardial performance index (MPI) and myocardial performance index by tissue Doppler. Bland-Altman plot for conventional myocardial performance index (MPI) by pulsed wave Doppler and myocardial performance index by tissue Doppler TDMPI of right ventricle (upper left) and left ventricle (lower left) in the athletes group and the right ventricle (upper right) and left ventricle (lower left) in the sedentary group. The mean value of the two methods is displayed on the x-axis and the difference between the methods is displayed on the y-axis. The two dotted lines represent a 95% confidence interval and the solid line represents the mean difference between the measurements

The Bland and Altman analysis illustrated in Fig. 2 shows a clinically important disagreement between the conventional MPI by PWD and MPI by TDI. Figure 2 demonstrates a comparison between MPIRV and TDMPIRV in athletes (the mean difference was 0.21 and 95% limits of agreement from −0.08 to 0.29) and in the sedentary controls MPIRV compared to TDMPIRV showed a mean difference of 0.25 (95% limits of agreement from −0.018 to 0.50). A comparison between MPILV and TDMPILV in athletes (the mean difference was 0.12 and 95% limits of agreement from −0.06 to 0.29) and in the sedentary controls MPILV compared to TDMPILV showed a mean difference of 0.17 (95% limits of agreement from −0.015 to 0.35).

We found no statistically significant correlation between the two methods measured in both RV (*r* = −0.027) and LV (*r* = 0.12) in the athletes group and RV (*r* = 0.20) and LV (*r* = 0.30) in the sedentary controls.

## Discussion

This is the first report, to our knowledge, on MPI in female athletes and also the first to measure MPI in both LV and RV. This study has demonstrated that there is no significant difference in conventional MPI by PWD and TDMPI between female elite team-handball players and sedentary controls. We have also shown that conventional MPI by PWD is significantly higher in LV compared to RV in both groups.

Team-handball is classified as a highly dynamic and moderate static sport, thus combining static and dynamic training, which has more morphological/structural effects on the heart compared to other sports [4]. The cardiac dimensions of our study population have previously been examined by Malmgren and co-workers [27], who showed a significant enlargement of cardiac dimensions in female elite team-handball players compared to sedentary controls. Thus, showing that cardiac remodelling appears in elite female handball players as it does in athletes practicing endurance or team game sports [27]. Hence, our findings are supported in the study of Dzudie et al. [3] that included 21 male team-handball players and 21 male controls showing morphological changes between the group of athletes compared to the control group but no difference in the LV diastolic function or ejection fraction (EF) between the two groups. In the meta-analysis by Pluim et al. [1] no relation between cardiac geometry and cardiac systolic and diastolic function could be found in athletes. Furthermore, no differences in cardiac systolic and diastolic function between athletes and sedentary controls were demonstrated [1]. Additionally, Butz and co-workers [28] studied the morphologic cardiac changes by echocardiography in 100 male professional handball players and reported a degree of hypertrophy and increased LV mass and end diastolic diameter in their study population [28]. However, they could not show any differences in diastolic function, nor in systolic or early diastolic velocities. Hence the results of the mentioned studies are similar to ours showing no significant difference in MPI between athletes and controls.

This current study did not show any difference in MPILV nor MPIRV between the two groups, which is in disagreement with the study by Kasikcioglu et al. [29] that involved 52 male athletes whereas 30 athletes were runners and 32 wrestlers, and a group of 43 sedentary controls. The study stated a significant difference in MPIRV between the athletes and the controls, showing a lower MPI at the RV of athletes compared to controls. Moreover, the study of Tüzün and co-workers [30] that included 66 elite male athletes (36 sprinters and 30 endurance athletes) and 33 sedentary controls were able to demonstrate a significant difference in MPILV between athletes and controls. MPI was reported as 0.37 ± 0.07 in sprinters and 0.36 ± 0.05 in endurance athletes and these results corresponds to our value of MPILV in athletes, which was reported as 0.35 ± 0.07. However, the value of MPI in the sedentary controls in both studies mentioned [29, 30] is much higher and does not correspond to ours. Also, their results does not correspond to earlier studies where the normal range for MPI was reported as 0.34 ± 0.04 in healthy volunteers by Moller et al. [31]. Previous studies have reported more effect on male athlete’s heart compared to females [6, 7]. Furthermore, this present study consisted of a lower number of participants compared to the study by Kasikcioglu et al. [29], which may be a limitation in the possibility of reporting a significant difference between the two groups. Besides, the male athletes in the mentioned study [29] trained as a minimum 10 h per week for at least 8 years. The athletes in our study had been training at elite level from 0 to 8 years. The significant difference in RV in the male athletes could have been caused by training for a longer period of time compared to the female athletes of this current study.

In both athletes and sedentary controls there is a significant difference in MPI by PWD between LV and RV. This difference may exist due to the different shape and geometry between the LV and the RV. The size of the RV is about two-thirds the size of the LV [32] and the RV wall is thinner (2–5 mm) compared to the wall of the LV (7–11 mm) [33]. Also, the LV has a twisting and rotating motion when contracting, while the twisting and rotating motion does not contribute to the contraction of the RV [34]. Besides, the pressure in the RV reaches an early peak compared to the LV and also a rapid decline in pressure. This is due to the low pulmonary artery diastolic pressure that the RV needs to exceed while contracting and therefore the ICT is shorter in RV compared to LV. The filling in RV starts before the LV and finishes after and also the IRT is shorter in RV compared to LV [34]. All of these different characters of LV and RV may be a reason of the significant difference between the ventricles seen in MPI by PWD. However, even though there are differences between the ventricles, they still generally pump the same effective stroke volume [34].

When TDMPI parameters were compared to one another in each group separately, all comparisons showed a significant difference in the sedentary control group. Although in the athletes group the TDMPIlm compared to TDMPIlt did not show any significant difference, neither did mTDMPIm/s compared to mTDMPIt/s. As seen in Table 3 the *p*-values in both groups follow the same pattern, whereas the *p*-value of TDMPIlm compared to TDMPIlt and mTDMPIm/s compared to mTDMPIt/s shows a weak significance in the sedentary controls. Consequently, since all other result were similar between both groups as well as the fact that all participants are young healthy subjects, they were all merged as a group to make the same comparisons. The results of this analysis are presented in Table 4 showing a significant difference between all TDMPI parameters. This indicates that TDMPI measured at the mitral, tricuspid and septal annulus is significantly different in all young and healthy subjects. These findings are similar to our findings presented from the conventional MPI by PWD that showed a difference in MPI between the two ventricles. Both LV and RV affect the septal annulus and therefore a significant difference is seen when comparing mTDMPIm/s and mTDMPIt/s. The different characters mentioned previously between the ventricles, also support these results showing a difference between LV and RV. The study by Rojo et al. [18] that included 77 patients with a previous myocardial infarction and a control group of 20 healthy young subjects, TDMPI was measured at septal annulus and lateral mitral annulus and additionally a mean value between those two measurements was calculated. In the healthy control group TDMPIlm compared to TDMPIs did not show any significant difference while in the present study there is a significant difference. The current study has a larger number of subjects included, which may be an explanation of that.

The results of this study have not been adjusted for age even though there was a significant difference in age between the athletes and the sedentary controls. Normally, the vascular stiffening is increased from the age of 30 and onward in both male and females [35]. The stiffening mostly affects the diastolic function of the LV, whereas the early diastolic filling rate decreases after the age of 20, so that by the age of 80, the early diastolic filling rate is declined to nearly 50% [35]. Since both groups are healthy young people, mostly in their early twenties, the adjustment for age was not considered to be of any significance.

The HR of athletes was significantly lower compared to the sedentary controls, but no adjustment for HR was made in this study. The reason is that MPI has been established to be independent of HR in a study by Tei et al. [11] consisting of 37 normal subjects and 26 patients with primary pulmonary hypertension. In that study, HR was correlated with each Doppler parameter in normal subjects and patients with primary pulmonary hypertension. The results showed a significant correlation between HR and ICT, IRT and ET. Though, there was no significant association between HR and MPI [11].

Keser et al. [15] showed a good correlation between the two methods measuring MPI and suggested the use of TDI when measuring MPI. This is due to the superiority of the methods’ sensitivity in not being affected by HR fluctuations. Also Harada et al. [17] showed very good correlation between the two methods. However, more recent studies have presented moderate to low correlation between the conventional way of measuring MPI and the modified method by TDI. Rojo et al. [18] suggested that the modified MPI by TDI is not possible to be used as an alternative to the conventional MPI by PWD due to the weak correlation between the two methods. The present study consisting of a higher number of participants compared to previously mentioned studies did not show any correlation between the two methods of measuring MPI and the agreement between the methods was shown to be low. Our findings are similar to those of Rojo et al. [18] that showed poor agreement between the methods due to longer systolic intervals and shorter diastolic intervals when measuring with TDI.

Furthermore, the Bland-Altman plots in Fig. 2 shows that the TDI measurements always show higher MPI values compared to measuring with PWD. These findings can be supported by the fact that the upper reference limit of MPI by PWD is 0.40, while the upper reference limit of TDMPI is 0.55 [26]. The difference between the two methods is that TDMPI is measured in one cardiac cycle, while the inflow and outflow velocities in left ventricle outflow tract and right ventricle outflow tract measured by the PWD are not possible to measure from the same cardiac cycle [26]. This might explain that no correlation was seen between the two methods.

Moreover, newer echocardiographic methods exist to evaluate LV and RV function in athletes. Vitarelli and co-workers [36] used TDI, speckle-tracking imaging (STI) and three-dimensional echocardiography to assess systemic ventricular-vascular function and RV function in athletes. They showed that ventricular and vascular response in athletes underlie different adaptations of ventricular volumes and arterial stiffness. STI was also used by Oxborough et al. [37] to estimate RV structure and function in ultra-endurance athletes and determine whether changes in the RV are correlated with alterations in LV function. The study could demonstrate that there is a RV dilatation and dysfunction in the recovery after an ultramarathon. Likewise LV systolic and diastolic function also is reduced after an ultramarathon which is believed to be a result of a combination of essential reduction in function and RV interaction. Additionally, Knackstedt et al. [38] used STI in former world class swimmers to evaluate the cardiac function, employing longitudinal strain and circumferential strain. The results of their study are in concordance with ours, showing that there is no definite LV or RV dysfunction in athletes using modern imaging modalities.

## Limitations

The limitation of measuring MPI in the conventional way by PWD is that the inflow and outflow velocities are measured separately. The reproducibility of the method is therefore reduced and is also affected by fluctuations in the HR during the examination. This makes the calculation of the index more complex, because several cardiac cycles have to be obtained to average the measurements.

## Conclusion

The global function of the LV and RV measured by MPI with PWD and TDI is similar in female elite team-handball players compared to sedentary controls. Conversely, both MPI by PWD and TDMPI shows a significant difference between LV and RV, whereas LV has higher MPI compared to RV. However, the agreement and correlation between conventional methods of measuring MPI by PWD compared to TDMPI is not good and therefore TDMPI should not be used interchangeably with MPI by PWD.

## Abbreviations

BMI: Body Mass Index
BSA: Body surface area
EF: Ejection fraction
ET: Ejection time
HR: Heart rate
ICT: Isovolumic contraction time
IRT: Isovolumic relaxation time
LV: Left ventricle
MPI: Myocardial performance index
MPI_LV: Myocardial performance index of left ventricle
MPI_RV: Myocardial performance index of right ventricle
MRI: Magnetic resonance imaging
mTDMPI_m/s: mean value of myocardial performance index by tissue Doppler measured at lateral mitral annulus and septal annulus
mTDMPI_t/s: mean value of myocardial performance index by tissue Doppler measured at lateral tricuspid annulus and septal annulus
PWD: Pulsed wave Doppler
RV: Right ventricle
RVET: Right ventricular ejection time
SD: Standard deviation
STI: Speckle tracking imaging
SV: Stroke volume
TDI: Tissue Doppler imaging
TDMPI: Myocardial performance index by tissue Doppler
TDMPI_lm: Myocardial performance index by tissue Doppler at lateral mitral annulus
TDMPI_lt: Myocardial performance index by tissue Doppler at lateral tricuspid annulus
TDMPI_s: Myocardial performance index by tissue Doppler at septal annulus
tET: Ejection time by tissue Doppler
tICT: Isovolumic contraction time by tissue Doppler
tIRT: Isovolumic relaxation time by tissue Doppler

## References

[1] Pluim BM, Zwinderman AH, van der Laarse A, van der Wall EE (2000). The athlete’s heart. A meta-analysis of cardiac structure and function. Circulation.

[2] Wernstedt P, Sjostedt C, Ekman I, Du H, Thuomas KA, Areskog NH (2002). Adaptation of cardiac morphology and function to endurance and strength training. A comparative study using MR imaging and echocardiography in males and females. Scand J Med Sci Sports.

[3] Dzudie A, Menanga A, Hamadou B, Kengne AP, Atchou G, Kingue S (2007). Ultrasonographic study of left ventricular function at rest in a group of highly trained black African handball players. Eur J Echocardiogr.

[4] Mitchell JH, Haskell W, Snell P, Van Camp SP (2005). Task Force 8: classification of sports. J Am Coll Cardiol.

[5] Pelliccia A, Maron BJ, Di Paolo FM, Biffi A, Quattrini FM, Pisicchio C (2005). Prevalence and clinical significance of left atrial remodeling in competitive athletes. J Am Coll Cardiol.

[6] Sharma S (2003). Athlete's heart--effect of age, sex, ethnicity and sporting discipline. Exp Physiol.

[7] Pelliccia A, Culasso F, Di Paolo FM, Maron BJ (1999). Physiologic left ventricular cavity dilatation in elite athletes. Ann Intern Med.

[8] Pelliccia A, Maron BJ, Spataro A, Proschan MA, Spirito P (1991). The upper limit of physiologic cardiac hypertrophy in highly trained elite athletes. N Engl J Med.

[9] Barbier J, Ville N, Kervio G, Walther G, Carre F (2006). Sports-specific features of athlete's heart and their relation to echocardiographic parameters. Herz.

[10] Miller D, Farah MG, Liner A, Fox K, Schluchter M, Hoit BD (2004). The relation between quantitative right ventricular ejection fraction and indices of tricuspid annular motion and myocardial performance. J Am Soc Echocardiogr.

[11] Tei C, Dujardin KS, Hodge DO, Bailey KR, McGoon MD, Tajik AJ (1996). Doppler echocardiographic index for assessment of global right ventricular function. J Am Soc Echocardiogr.

[12] Pagourelias ED, Kouidi E, Efthimiadis GK, Deligiannis A, Geleris P, Vassilikos V (2013). Right atrial and ventricular adaptations to training in male Caucasian athletes: an echocardiographic study. J Am Soc Echocardiogr.

[13] Su HM, Lin TH, Voon WC, Lee KT, Chu CS, Lai WT (2006). Differentiation of left ventricular diastolic dysfunction, identification of pseudonormal/restrictive mitral inflow pattern and determination of left ventricular filling pressure by Tei index obtained from tissue Doppler echocardiography. Echocardiography..

[14] Tei C, Nishimura RA, Seward JB, Tajik AJ (1997). Noninvasive Doppler-derived myocardial performance index: correlation with simultaneous measurements of cardiac catheterization measurements. J Am Soc Echocardiogr.

[15] Keser N, Yildiz S, Kurtog N, Dindar I (2005). Modified TEI index: a promising parameter in essential hypertension?. Echocardiography..

[16] Tei C, Ling LH, Hodge DO, Bailey KR, Oh JK, Rodeheffer RJ (1995). New index of combined systolic and diastolic myocardial performance: a simple and reproducible measure of cardiac function--a study in normals and dilated cardiomyopathy. J Cardiol.

[17] Harada K, Tamura M, Toyono M, Yasuoka K (2002). Comparison of the right ventricular Tei index by tissue Doppler imaging to that obtained by pulsed Doppler in children without heart disease. Am J Cardiol.

[18] Rojo EC, Rodrigo JL, Perez de Isla L, Almeria C, Gonzalo N, Aubele A (2006). Disagreement between tissue Doppler imaging and conventional pulsed wave Doppler in the measurement of myocardial performance index. Eur J Echocardiogr.

[19] Gaibazzi N, Petrucci N, Ziacchi V (2005). Left ventricle myocardial performance index derived either by conventional method or mitral annulus tissue-Doppler: a comparison study in healthy subjects and subjects with heart failure. J Am Soc Echocardiogr.

[20] Carluccio E, Biagioli P, Alunni G, Murrone A, Zuchi C, Biscottini E (2012). Improvement of myocardial performance (Tei) index closely reflects intrinsic improvement of cardiac function: assessment in revascularized hibernating myocardium. Echocardiography.

[21] Tei C, Dujardin KS, Hodge DO, Kyle RA, Tajik AJ, Seward JB (1996). Doppler index combining systolic and diastolic myocardial performance: clinical value in cardiac amyloidosis. J Am Coll Cardiol.

[22] Vonk MC, Sander MH, van den Hoogen FH, van Riel PL, Verheugt FW, van Dijk AP (2007). Right ventricle Tei-index: a tool to increase the accuracy of non-invasive detection of pulmonary arterial hypertension in connective tissue diseases. Eur J Echocardiogr.

[23] Arnlov J, Lind L, Andren B, Riserus U, Berglund L, Lithell H (2005). A Doppler-derived index of combined left ventricular systolic and diastolic function is an independent predictor of cardiovascular mortality in elderly men. Am Heart J.

[24] Lind L, Andren B, Arnlov J. The Doppler-derived myocardial performance index is determined by both left ventricular systolic and diastolic function as well as by afterload and left ventricular mass. Echocardiography. 2005;22(3):211–6. 10.1111/j.0742-2822.2005.03175.x 15725155

[25] Du Bois D, Du Bois EF (1989). A formula to estimate the approximate surface area if height and weight be known. 1916. Nutrition.

[26] Rudski LG, Lai WW, Afilalo J, Hua L, Handschumacher MD, Chandrasekaran K (2010). Guidelines for the echocardiographic assessment of the right heart in adults: a report from the American Society of Echocardiography endorsed by the European Association of Echocardiography, a registered branch of the European Society of Cardiology, and the Canadian Society of Echocardiography. J Am Soc Echocardiogr.

[27] Malmgren A, Dencker M, Stagmo M, Gudmundsson P. Cardiac dimensions and function in female handball players. J Sports Med Phys Fitness. 2015;55(4):320–8. 25600906

[28] Butz T, van Buuren F, Mellwig KP, Langer C, Oldenburg O, Treusch KA (2010). Systolic and early diastolic left ventricular velocities assessed by tissue Doppler imaging in 100 top-level handball players. Eur J Cardiovasc Prev Rehabil.

[29] Kasikcioglu E, Oflaz H, Akhan H, Kayserilioglu A (2005). Right ventricular myocardial performance index and exercise capacity in athletes. Heart Vessel.

[30] Tuzun N, Ergun M, Alioglu E, Edem E, Tengiz I, Aytemiz F (2015). TEI Index in elite sprinters and endurance athletes. J Sports Med Phys Fitness.

[31] Moller JE, Poulsen SH, Egstrup K (1999). Effect of preload alternations on a new Doppler echocardiographic index of combined systolic and diastolic performance. J Am Soc Echocardiogr.

[32] Bleeker GB, Steendijk P, Holman ER, Yu CM, Breithardt OA, Kaandorp TA (2006). Assessing right ventricular function: the role of echocardiography and complementary technologies. Heart.

[33] Auger DA, Zhong X, Epstein FH, Spottiswoode BS (2012). Mapping right ventricular myocardial mechanics using 3D cine DENSE cardiovascular magnetic resonance. J Cardiovasc Magn Reson.

[34] Haddad F, Hunt SA, Rosenthal DN, Murphy DJ (2008). Right ventricular function in cardiovascular disease, part I: anatomy, physiology, aging, and functional assessment of the right ventricle. Circulation.

[35] Hollingsworth KG, Blamire AM, Keavney BD, Macgowan GA (2012). Left ventricular torsion, energetics, and diastolic function in normal human aging. Am J Physiol Heart Circ Physiol.

[36] Vitarelli A, Capotosto L, Placanica G, Caranci F, Pergolini M, Zardo F (2013). Comprehensive assessment of biventricular function and aortic stiffness in athletes with different forms of training by three-dimensional echocardiography and strain imaging. Eur Heart J Cardiovasc Imaging.

[37] Oxborough D, Shave R, Warburton D, Williams K, Oxborough A, Charlesworth S (2011). Dilatation and dysfunction of the right ventricle immediately after ultraendurance exercise: exploratory insights from conventional two-dimensional and speckle tracking echocardiography. Circ Cardiovasc Imaging.

[38] Knackstedt C, Hildebrandt U, Schmidt K, Syrocki L, Lang A, Bjarnason-Wehrens B (2015). Analysis of right and left ventricular deformation in former world class swimmers: evaluation using speckle tracking. J Sports Med Phys Fitness.

